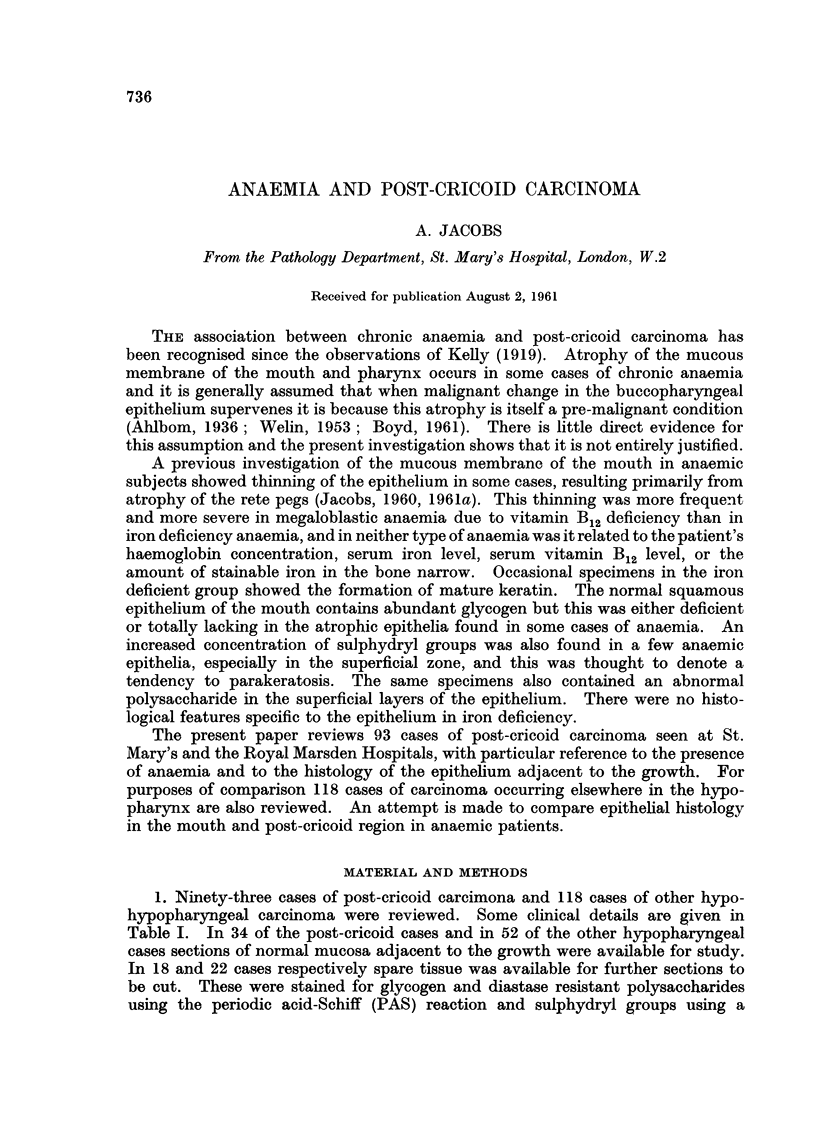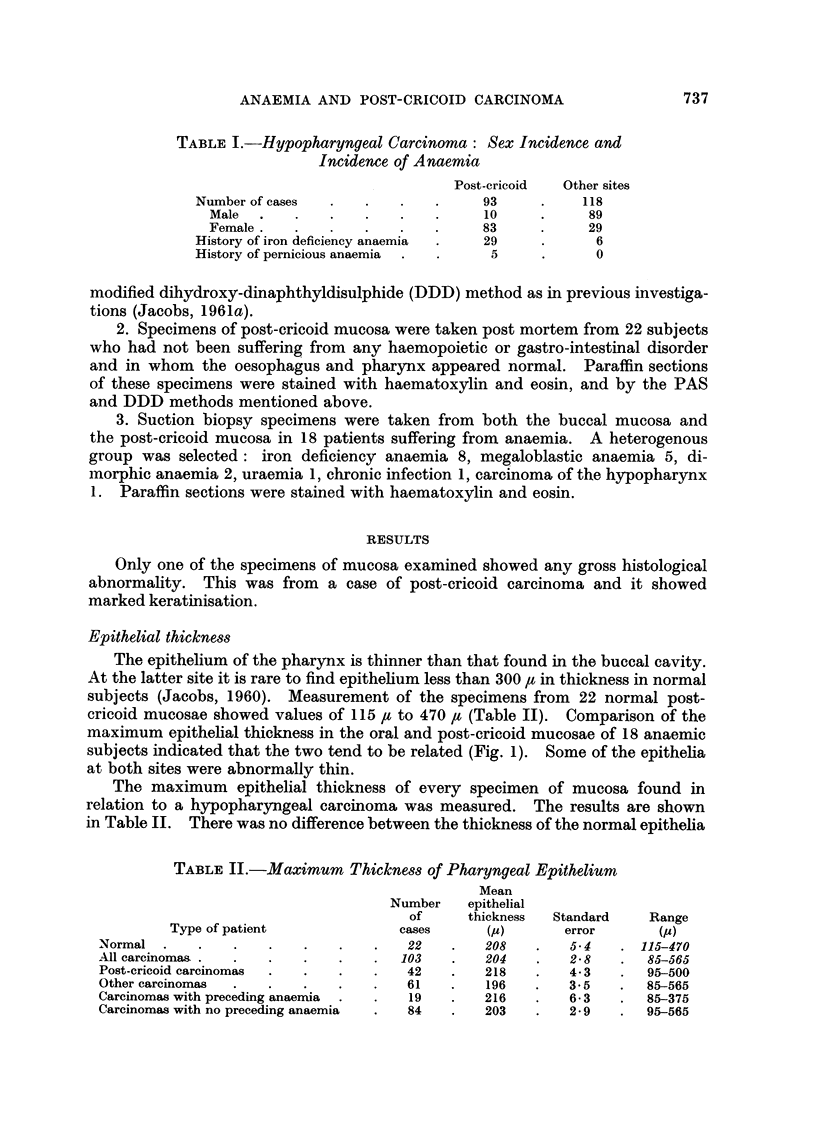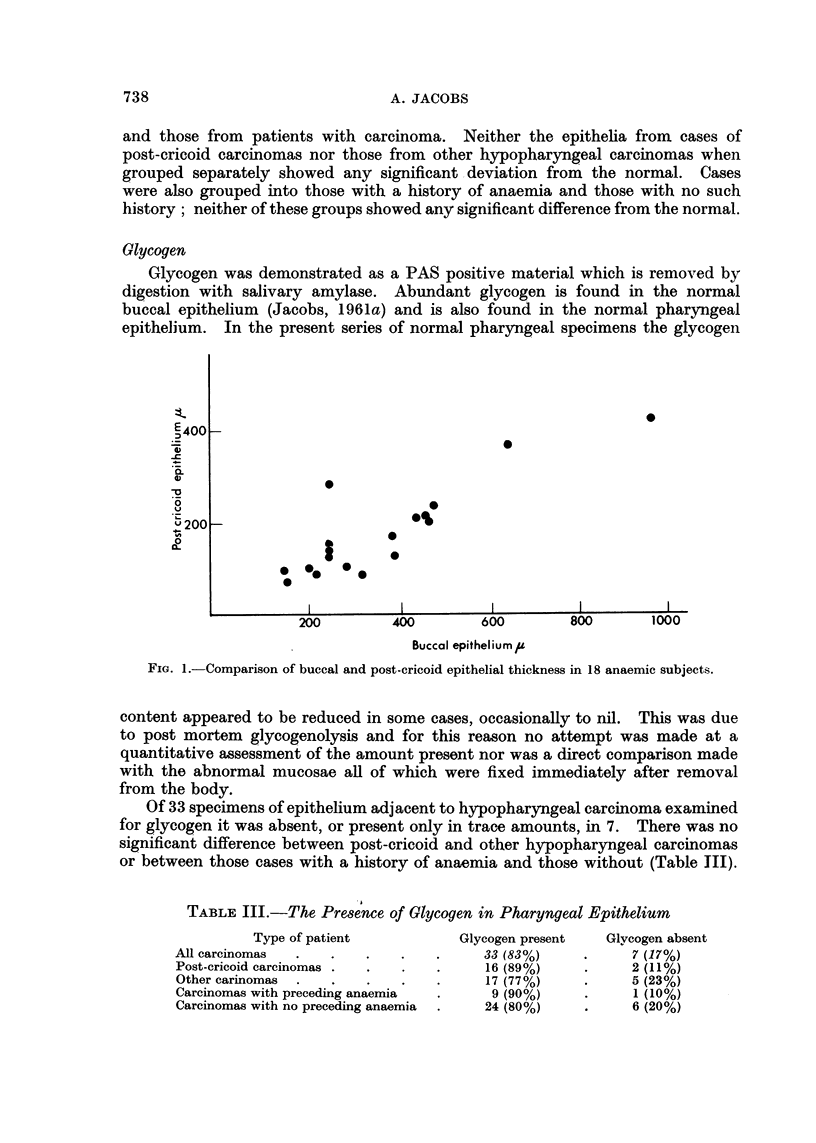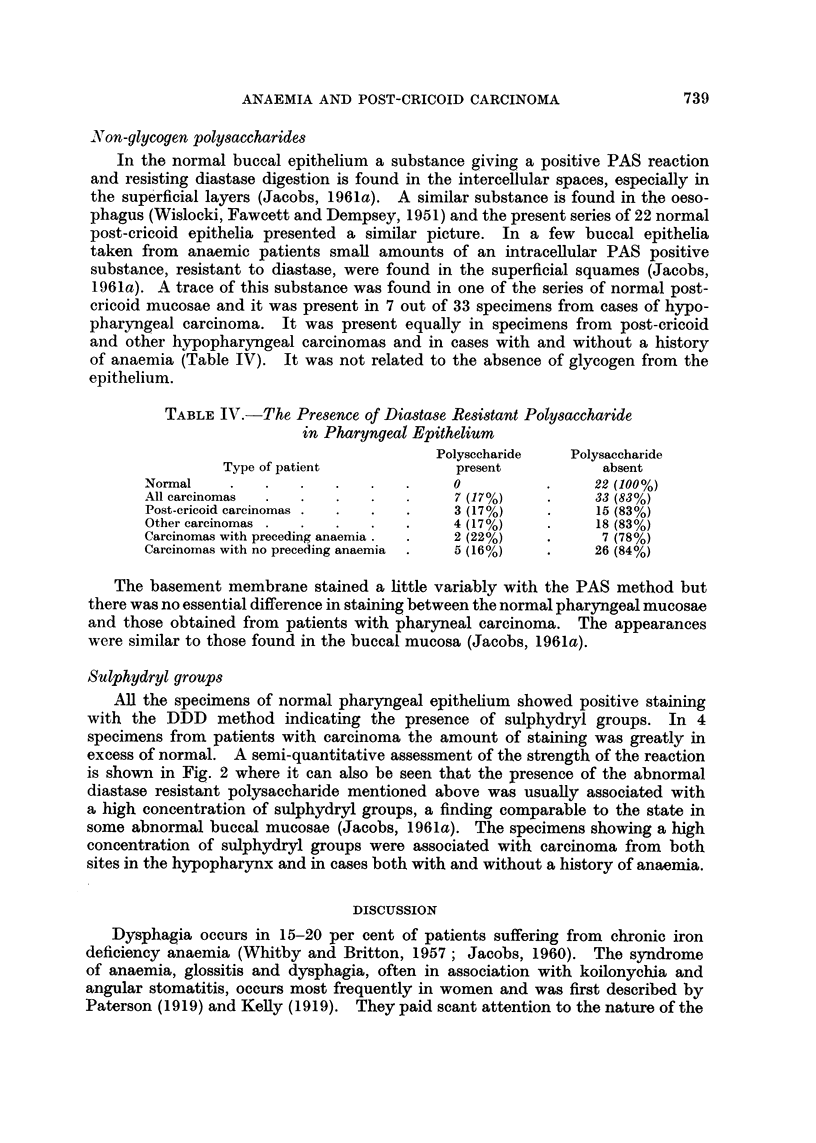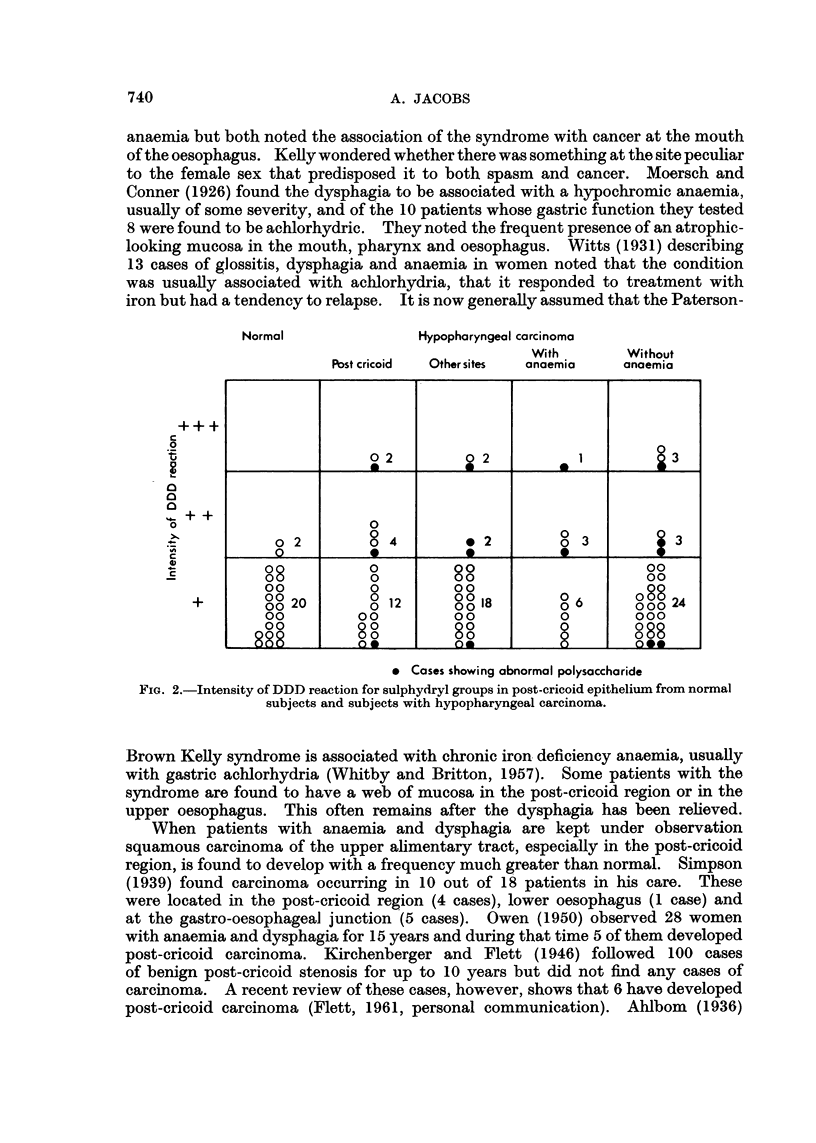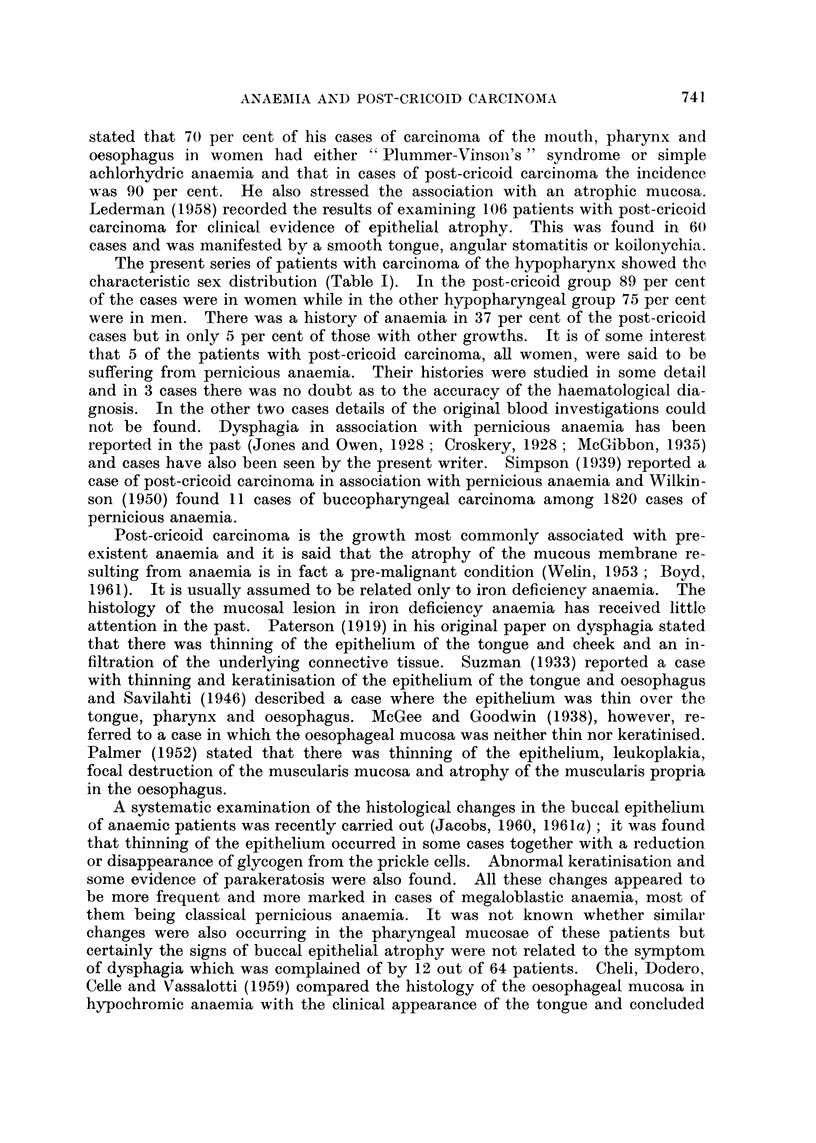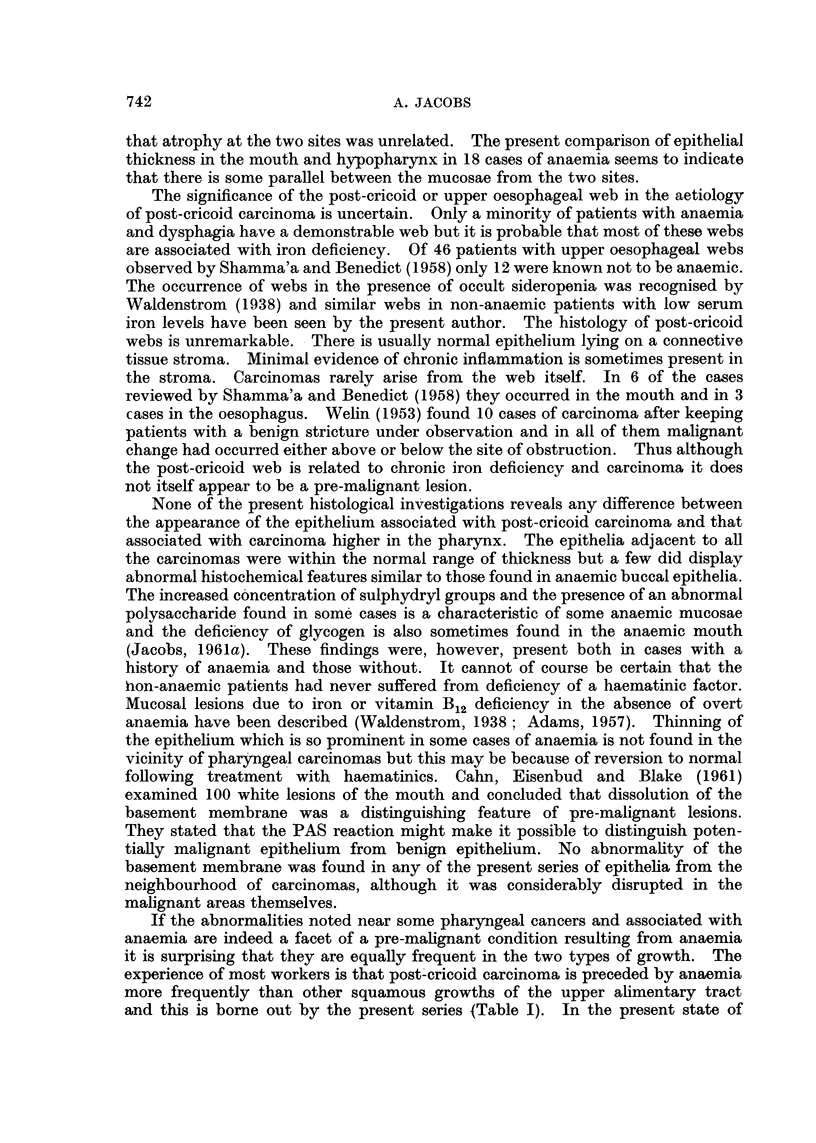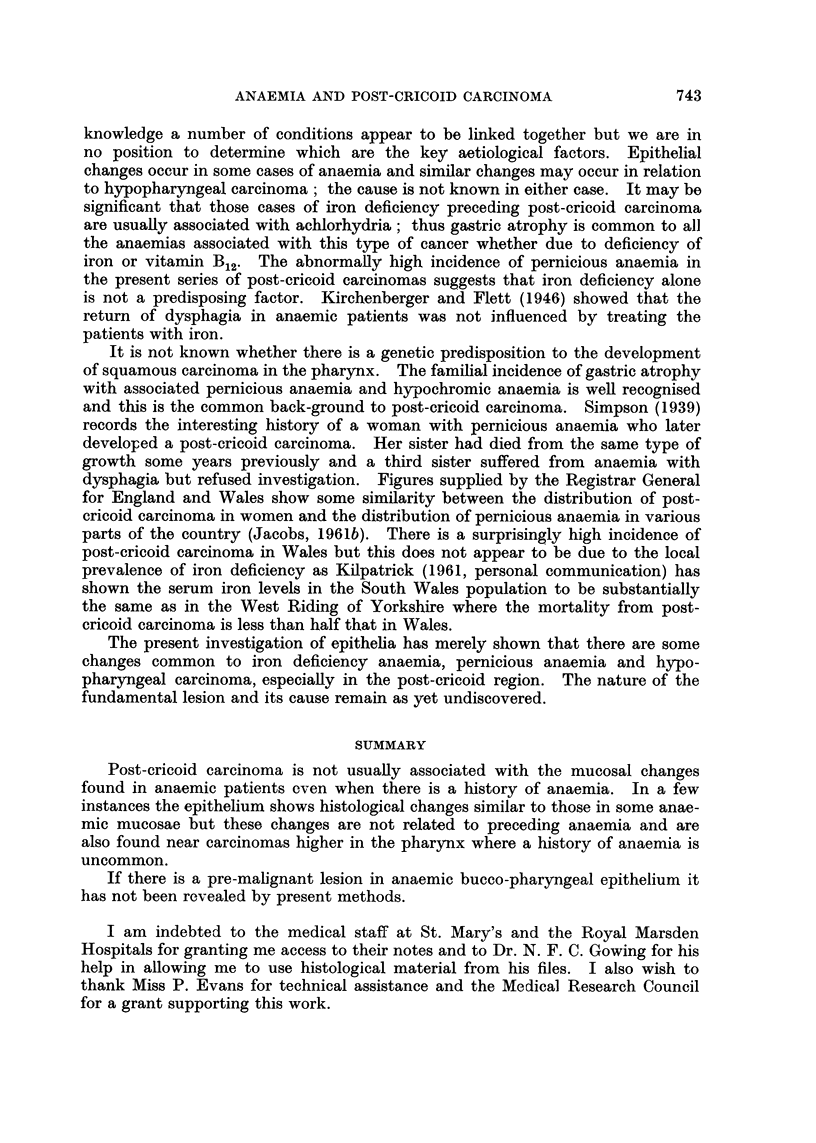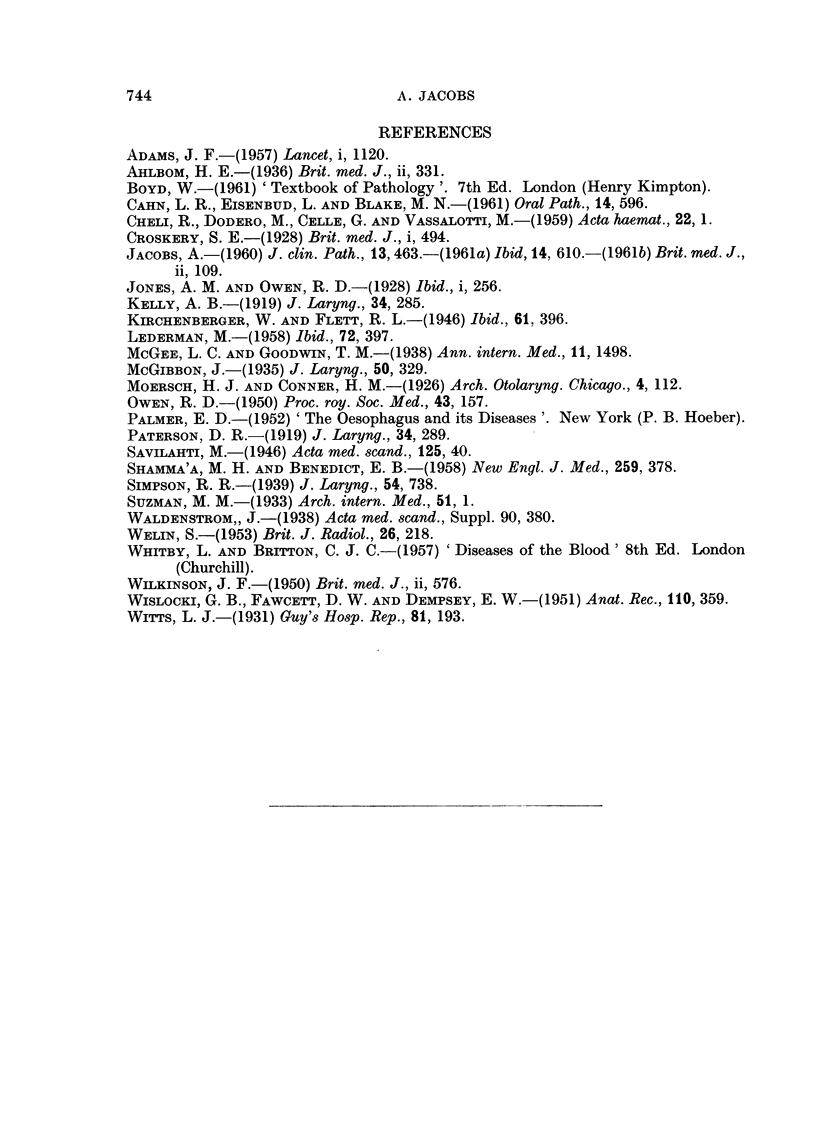# Anaemia and Post-Cricoid Carcinoma

**DOI:** 10.1038/bjc.1961.84

**Published:** 1961-12

**Authors:** A. Jacobs


### ANAEMIA AND POST-CRICOID CARCINOMA

A. JACOBS

*From the Pathology Department, St. Mary's Hospital, London, W.2*

Received for publication August 2, 1961

THE association between chronic anaemia and post-cricoid carcinoma has been recognised since the observations of Kelly (1919). Atrophy of the mucous membrane of the mouth and pharynx occurs in some cases of chronic anaemia and it is generally assumed that when malignant change in the buccopharyngeal epithelium supervenes it is because this atrophy is itself a pre-malignant condition (Ahlbom, 1936 ; Welin, 1953 ; Boyd, 1961). There is little direct evidence for this assumption and the present investigation shows that it is not entirely justified.

A previous investigation of the mucous membrane of the mouth in anaemic subjects showed thinning of the epithelium in some cases, resulting primarily from atrophy of the rete pegs (Jacobs, 1960, 1961*a*). This thinning was more frequent and more severe in megaloblastic anaemia due to vitamin $B_{12}$ deficiency than in iron deficiency anaemia, and in neither type of anaemia was it related to the patient's haemoglobin concentration, serum iron level, serum vitamin $B_{12}$ level, or the amount of stainable iron in the bone narrow. Occasional specimens in the iron deficient group showed the formation of mature keratin. The normal squamous epithelium of the mouth contains abundant glycogen but this was either deficient or totally lacking in the atrophic epithelia found in some cases of anaemia. An increased concentration of sulphydryl groups was also found in a few anaemic epithelia, especially in the superficial zone, and this was thought to denote a tendency to parakeratosis. The same specimens also contained an abnormal polysaccharide in the superficial layers of the epithelium. There were no histological features specific to the epithelium in iron deficiency.

The present paper reviews 93 cases of post-cricoid carcinoma seen at St. Mary's and the Royal Marsden Hospitals, with particular reference to the presence of anaemia and to the histology of the epithelium adjacent to the growth. For purposes of comparison 118 cases of carcinoma occurring elsewhere in the hypopharynx are also reviewed. An attempt is made to compare epithelial histology in the mouth and post-cricoid region in anaemic patients.

### MATERIAL AND METHODS

1. Ninety-three cases of post-cricoid carcimona and 118 cases of other hypohypopharyngeal carcinoma were reviewed. Some clinical details are given in Table I. In 34 of the post-cricoid cases and in 52 of the other hypopharyngeal cases sections of normal mucosa adjacent to the growth were available for study. In 18 and 22 cases respectively spare tissue was available for further sections to be cut. These were stained for glycogen and diastase resistant polysaccharides using the periodic acid-Schiff (PAS) reaction and sulphydryl groups using a

TABLE I.—*Hypopharyngeal Carcinoma : Sex Incidence and Incidence of Anaemia*

| | Post-cricoid | Other sites |
|---|---|---|
| Number of cases | 93 | 118 |
| Male | 10 | 89 |
| Female | 83 | 29 |
| History of iron deficiency anaemia | 29 | 6 |
| History of pernicious anaemia | 5 | 0 |

modified dihydroxy-dinaphthyldisulphide (DDD) method as in previous investigations (Jacobs, 1961*a*).

2. Specimens of post-cricoid mucosa were taken post mortem from 22 subjects who had not been suffering from any haemopoietic or gastro-intestinal disorder and in whom the oesophagus and pharynx appeared normal. Paraffin sections of these specimens were stained with haematoxylin and eosin, and by the PAS and DDD methods mentioned above.

3. Suction biopsy specimens were taken from both the buccal mucosa and the post-cricoid mucosa in 18 patients suffering from anaemia. A heterogenous group was selected : iron deficiency anaemia 8, megaloblastic anaemia 5, dimorphic anaemia 2, uraemia 1, chronic infection 1, carcinoma of the hypopharynx 1. Paraffin sections were stained with haematoxylin and eosin.

### RESULTS

Only one of the specimens of mucosa examined showed any gross histological abnormality. This was from a case of post-cricoid carcinoma and it showed marked keratinisation.

*Epithelial thickness*

The epithelium of the pharynx is thinner than that found in the buccal cavity. At the latter site it is rare to find epithelium less than 300 $\mu$ in thickness in normal subjects (Jacobs, 1960). Measurement of the specimens from 22 normal post-cricoid mucosae showed values of 115 $\mu$ to 470 $\mu$ (Table II). Comparison of the maximum epithelial thickness in the oral and post-cricoid mucosae of 18 anaemic subjects indicated that the two tend to be related (Fig. 1). Some of the epithelia at both sites were abnormally thin.

The maximum epithelial thickness of every specimen of mucosa found in relation to a hypopharyngeal carcinoma was measured. The results are shown in Table II. There was no difference between the thickness of the normal epithelia

TABLE II.—*Maximum Thickness of Pharyngeal Epithelium*

| Type of patient | Number of cases | Mean epithelial thickness ($\mu$) | Standard error | Range ($\mu$) |
|---|---|---|---|---|
| Normal | *22* | *208* | *5·4* | *115–470* |
| All carcinomas | *103* | *204* | *2·8* | *85–565* |
| Post-cricoid carcinomas | 42 | 218 | 4·3 | 95–500 |
| Other carcinomas | 61 | 196 | 3·5 | 85–565 |
| Carcinomas with preceding anaemia | 19 | 216 | 6·3 | 85–375 |
| Carcinomas with no preceding anaemia | 84 | 203 | 2·9 | 95–565 |

and those from patients with carcinoma. Neither the epithelia from cases of post-cricoid carcinomas nor those from other hypopharyngeal carcinomas when grouped separately showed any significant deviation from the normal. Cases were also grouped into those with a history of anaemia and those with no such history ; neither of these groups showed any significant difference from the normal.

*Glycogen*

Glycogen was demonstrated as a PAS positive material which is removed by digestion with salivary amylase. Abundant glycogen is found in the normal buccal epithelium (Jacobs, 1961*a*) and is also found in the normal pharyngeal epithelium. In the present series of normal pharyngeal specimens the glycogen

Fig. 1.—Comparison of buccal and post-cricoid epithelial thickness in 18 anaemic subjects.

content appeared to be reduced in some cases, occasionally to nil. This was due to post mortem glycogenolysis and for this reason no attempt was made at a quantitative assessment of the amount present nor was a direct comparison made with the abnormal mucosae all of which were fixed immediately after removal from the body.

Of 33 specimens of epithelium adjacent to hypopharyngeal carcinoma examined for glycogen it was absent, or present only in trace amounts, in 7. There was no significant difference between post-cricoid and other hypopharyngeal carcinomas or between those cases with a history of anaemia and those without (Table III).

Table III.—*The Presence of Glycogen in Pharyngeal Epithelium*

| Type of patient | Glycogen present | Glycogen absent |
|---|---|---|
| All carcinomas | *33 (83%)* | *7 (17%)* |
| Post-cricoid carcinomas | 16 (89%) | 2 (11%) |
| Other carinomas | 17 (77%) | 5 (23%) |
| Carcinomas with preceding anaemia | 9 (90%) | 1 (10%) |
| Carcinomas with no preceding anaemia | 24 (80%) | 6 (20%) |

*Non-glycogen polysaccharides*

In the normal buccal epithelium a substance giving a positive PAS reaction and resisting diastase digestion is found in the intercellular spaces, especially in the superficial layers (Jacobs, 1961*a*). A similar substance is found in the oesophagus (Wislocki, Fawcett and Dempsey, 1951) and the present series of 22 normal post-cricoid epithelia presented a similar picture. In a few buccal epithelia taken from anaemic patients small amounts of an intracellular PAS positive substance, resistant to diastase, were found in the superficial squames (Jacobs, 1961*a*). A trace of this substance was found in one of the series of normal post-cricoid mucosae and it was present in 7 out of 33 specimens from cases of hypopharyngeal carcinoma. It was present equally in specimens from post-cricoid and other hypopharyngeal carcinomas and in cases with and without a history of anaemia (Table IV). It was not related to the absence of glycogen from the epithelium.

TABLE IV.—*The Presence of Diastase Resistant Polysaccharide in Pharyngeal Epithelium*

| Type of patient | Polysccharide present | Polysaccharide absent |
|---|---|---|
| Normal | *0* | *22 (100%)* |
| All carcinomas | *7 (17%)* | *33 (83%)* |
| Post-cricoid carcinomas | 3 (17%) | 15 (83%) |
| Other carcinomas | 4 (17%) | 18 (83%) |
| Carcinomas with preceding anaemia | 2 (22%) | 7 (78%) |
| Carcinomas with no preceding anaemia | 5 (16%) | 26 (84%) |

The basement membrane stained a little variably with the PAS method but there was no essential difference in staining between the normal pharyngeal mucosae and those obtained from patients with pharyneal carcinoma. The appearances were similar to those found in the buccal mucosa (Jacobs, 1961*a*).

*Sulphydryl groups*

All the specimens of normal pharyngeal epithelium showed positive staining with the DDD method indicating the presence of sulphydryl groups. In 4 specimens from patients with carcinoma the amount of staining was greatly in excess of normal. A semi-quantitative assessment of the strength of the reaction is shown in Fig. 2 where it can also be seen that the presence of the abnormal diastase resistant polysaccharide mentioned above was usually associated with a high concentration of sulphydryl groups, a finding comparable to the state in some abnormal buccal mucosae (Jacobs, 1961*a*). The specimens showing a high concentration of sulphydryl groups were associated with carcinoma from both sites in the hypopharynx and in cases both with and without a history of anaemia.

### DISCUSSION

Dysphagia occurs in 15–20 per cent of patients suffering from chronic iron deficiency anaemia (Whitby and Britton, 1957; Jacobs, 1960). The syndrome of anaemia, glossitis and dysphagia, often in association with koilonychia and angular stomatitis, occurs most frequently in women and was first described by Paterson (1919) and Kelly (1919). They paid scant attention to the nature of the anaemia but both noted the association of the syndrome with cancer at the mouth of the oesophagus. Kelly wondered whether there was something at the site peculiar to the female sex that predisposed it to both spasm and cancer. Moersch and Conner (1926) found the dysphagia to be associated with a hypochromic anaemia, usually of some severity, and of the 10 patients whose gastric function they tested 8 were found to be achlorhydric. They noted the frequent presence of an atrophic-looking mucosa in the mouth, pharynx and oesophagus. Witts (1931) describing 13 cases of glossitis, dysphagia and anaemia in women noted that the condition was usually associated with achlorhydria, that it responded to treatment with iron but had a tendency to relapse. It is now generally assumed that the Paterson-

FIG. 2.—Intensity of DDD reaction for sulphydryl groups in post-cricoid epithelium from normal subjects and subjects with hypopharyngeal carcinoma.

Brown Kelly syndrome is associated with chronic iron deficiency anaemia, usually with gastric achlorhydria (Whitby and Britton, 1957). Some patients with the syndrome are found to have a web of mucosa in the post-cricoid region or in the upper oesophagus. This often remains after the dysphagia has been relieved.

When patients with anaemia and dysphagia are kept under observation squamous carcinoma of the upper alimentary tract, especially in the post-cricoid region, is found to develop with a frequency much greater than normal. Simpson (1939) found carcinoma occurring in 10 out of 18 patients in his care. These were located in the post-cricoid region (4 cases), lower oesophagus (1 case) and at the gastro-oesophageal junction (5 cases). Owen (1950) observed 28 women with anaemia and dysphagia for 15 years and during that time 5 of them developed post-cricoid carcinoma. Kirchenberger and Flett (1946) followed 100 cases of benign post-cricoid stenosis for up to 10 years but did not find any cases of carcinoma. A recent review of these cases, however, shows that 6 have developed post-cricoid carcinoma (Flett, 1961, personal communication). Ahlbom (1936)

stated that 70 per cent of his cases of carcinoma of the mouth, pharynx and oesophagus in women had either "Plummer-Vinson's" syndrome or simple achlorhydric anaemia and that in cases of post-cricoid carcinoma the incidence was 90 per cent. He also stressed the association with an atrophic mucosa. Lederman (1958) recorded the results of examining 106 patients with post-cricoid carcinoma for clinical evidence of epithelial atrophy. This was found in 60 cases and was manifested by a smooth tongue, angular stomatitis or koilonychia.

The present series of patients with carcinoma of the hypopharynx showed the characteristic sex distribution (Table I). In the post-cricoid group 89 per cent of the cases were in women while in the other hypopharyngeal group 75 per cent were in men. There was a history of anaemia in 37 per cent of the post-cricoid cases but in only 5 per cent of those with other growths. It is of some interest that 5 of the patients with post-cricoid carcinoma, all women, were said to be suffering from pernicious anaemia. Their histories were studied in some detail and in 3 cases there was no doubt as to the accuracy of the haematological diagnosis. In the other two cases details of the original blood investigations could not be found. Dysphagia in association with pernicious anaemia has been reported in the past (Jones and Owen, 1928; Croskery, 1928; McGibbon, 1935) and cases have also been seen by the present writer. Simpson (1939) reported a case of post-cricoid carcinoma in association with pernicious anaemia and Wilkinson (1950) found 11 cases of buccopharyngeal carcinoma among 1820 cases of pernicious anaemia.

Post-cricoid carcinoma is the growth most commonly associated with pre-existent anaemia and it is said that the atrophy of the mucous membrane resulting from anaemia is in fact a pre-malignant condition (Welin, 1953; Boyd, 1961). It is usually assumed to be related only to iron deficiency anaemia. The histology of the mucosal lesion in iron deficiency anaemia has received little attention in the past. Paterson (1919) in his original paper on dysphagia stated that there was thinning of the epithelium of the tongue and cheek and an infiltration of the underlying connective tissue. Suzman (1933) reported a case with thinning and keratinisation of the epithelium of the tongue and oesophagus and Savilahti (1946) described a case where the epithelium was thin over the tongue, pharynx and oesophagus. McGee and Goodwin (1938), however, referred to a case in which the oesophageal mucosa was neither thin nor keratinised. Palmer (1952) stated that there was thinning of the epithelium, leukoplakia, focal destruction of the muscularis mucosa and atrophy of the muscularis propria in the oesophagus.

A systematic examination of the histological changes in the buccal epithelium of anaemic patients was recently carried out (Jacobs, 1960, 1961*a*); it was found that thinning of the epithelium occurred in some cases together with a reduction or disappearance of glycogen from the prickle cells. Abnormal keratinisation and some evidence of parakeratosis were also found. All these changes appeared to be more frequent and more marked in cases of megaloblastic anaemia, most of them being classical pernicious anaemia. It was not known whether similar changes were also occurring in the pharyngeal mucosae of these patients but certainly the signs of buccal epithelial atrophy were not related to the symptom of dysphagia which was complained of by 12 out of 64 patients. Cheli, Dodero, Celle and Vassalotti (1959) compared the histology of the oesophageal mucosa in hypochromic anaemia with the clinical appearance of the tongue and concluded that atrophy at the two sites was unrelated. The present comparison of epithelial thickness in the mouth and hypopharynx in 18 cases of anaemia seems to indicate that there is some parallel between the mucosae from the two sites.

The significance of the post-cricoid or upper oesophageal web in the aetiology of post-cricoid carcinoma is uncertain. Only a minority of patients with anaemia and dysphagia have a demonstrable web but it is probable that most of these webs are associated with iron deficiency. Of 46 patients with upper oesophageal webs observed by Shamma'a and Benedict (1958) only 12 were known not to be anaemic. The occurrence of webs in the presence of occult sideropenia was recognised by Waldenstrom (1938) and similar webs in non-anaemic patients with low serum iron levels have been seen by the present author. The histology of post-cricoid webs is unremarkable. There is usually normal epithelium lying on a connective tissue stroma. Minimal evidence of chronic inflammation is sometimes present in the stroma. Carcinomas rarely arise from the web itself. In 6 of the cases reviewed by Shamma'a and Benedict (1958) they occurred in the mouth and in 3 cases in the oesophagus. Welin (1953) found 10 cases of carcinoma after keeping patients with a benign stricture under observation and in all of them malignant change had occurred either above or below the site of obstruction. Thus although the post-cricoid web is related to chronic iron deficiency and carcinoma it does not itself appear to be a pre-malignant lesion.

None of the present histological investigations reveals any difference between the appearance of the epithelium associated with post-cricoid carcinoma and that associated with carcinoma higher in the pharynx. The epithelia adjacent to all the carcinomas were within the normal range of thickness but a few did display abnormal histochemical features similar to those found in anaemic buccal epithelia. The increased concentration of sulphydryl groups and the presence of an abnormal polysaccharide found in some cases is a characteristic of some anaemic mucosae and the deficiency of glycogen is also sometimes found in the anaemic mouth (Jacobs, 1961*a*). These findings were, however, present both in cases with a history of anaemia and those without. It cannot of course be certain that the non-anaemic patients had never suffered from deficiency of a haematinic factor. Mucosal lesions due to iron or vitamin $B_{12}$ deficiency in the absence of overt anaemia have been described (Waldenstrom, 1938; Adams, 1957). Thinning of the epithelium which is so prominent in some cases of anaemia is not found in the vicinity of pharyngeal carcinomas but this may be because of reversion to normal following treatment with haematinics. Cahn, Eisenbud and Blake (1961) examined 100 white lesions of the mouth and concluded that dissolution of the basement membrane was a distinguishing feature of pre-malignant lesions. They stated that the PAS reaction might make it possible to distinguish potentially malignant epithelium from benign epithelium. No abnormality of the basement membrane was found in any of the present series of epithelia from the neighbourhood of carcinomas, although it was considerably disrupted in the malignant areas themselves.

If the abnormalities noted near some pharyngeal cancers and associated with anaemia are indeed a facet of a pre-malignant condition resulting from anaemia it is surprising that they are equally frequent in the two types of growth. The experience of most workers is that post-cricoid carcinoma is preceded by anaemia more frequently than other squamous growths of the upper alimentary tract and this is borne out by the present series (Table I). In the present state of knowledge a number of conditions appear to be linked together but we are in no position to determine which are the key aetiological factors. Epithelial changes occur in some cases of anaemia and similar changes may occur in relation to hypopharyngeal carcinoma ; the cause is not known in either case. It may be significant that those cases of iron deficiency preceding post-cricoid carcinoma are usually associated with achlorhydria ; thus gastric atrophy is common to all the anaemias associated with this type of cancer whether due to deficiency of iron or vitamin $B_{12}$. The abnormally high incidence of pernicious anaemia in the present series of post-cricoid carcinomas suggests that iron deficiency alone is not a predisposing factor. Kirchenberger and Flett (1946) showed that the return of dysphagia in anaemic patients was not influenced by treating the patients with iron.

It is not known whether there is a genetic predisposition to the development of squamous carcinoma in the pharynx. The familial incidence of gastric atrophy with associated pernicious anaemia and hypochromic anaemia is well recognised and this is the common back-ground to post-cricoid carcinoma. Simpson (1939) records the interesting history of a woman with pernicious anaemia who later developed a post-cricoid carcinoma. Her sister had died from the same type of growth some years previously and a third sister suffered from anaemia with dysphagia but refused investigation. Figures supplied by the Registrar General for England and Wales show some similarity between the distribution of post-cricoid carcinoma in women and the distribution of pernicious anaemia in various parts of the country (Jacobs, 1961*b*). There is a surprisingly high incidence of post-cricoid carcinoma in Wales but this does not appear to be due to the local prevalence of iron deficiency as Kilpatrick (1961, personal communication) has shown the serum iron levels in the South Wales population to be substantially the same as in the West Riding of Yorkshire where the mortality from post-cricoid carcinoma is less than half that in Wales.

The present investigation of epithelia has merely shown that there are some changes common to iron deficiency anaemia, pernicious anaemia and hypopharyngeal carcinoma, especially in the post-cricoid region. The nature of the fundamental lesion and its cause remain as yet undiscovered.

### SUMMARY

Post-cricoid carcinoma is not usually associated with the mucosal changes found in anaemic patients even when there is a history of anaemia. In a few instances the epithelium shows histological changes similar to those in some anaemic mucosae but these changes are not related to preceding anaemia and are also found near carcinomas higher in the pharynx where a history of anaemia is uncommon.

If there is a pre-malignant lesion in anaemic bucco-pharyngeal epithelium it has not been revealed by present methods.

I am indebted to the medical staff at St. Mary's and the Royal Marsden Hospitals for granting me access to their notes and to Dr. N. F. C. Gowing for his help in allowing me to use histological material from his files. I also wish to thank Miss P. Evans for technical assistance and the Medical Research Council for a grant supporting this work.

### REFERENCES

ADAMS, J. F.—(1957) *Lancet*, i, 1120.
AHLBOM, H. E.—(1936) *Brit. med. J.*, ii, 331.
BOYD, W.—(1961) ' Textbook of Pathology '. 7th Ed. London (Henry Kimpton).
CAHN, L. R., EISENBUD, L. AND BLAKE, M. N.—(1961) *Oral Path.*, **14**, 596.
CHELI, R., DODERO, M., CELLE, G. AND VASSALOTTI, M.—(1959) *Acta haemat.*, **22**, 1.
CROSKERY, S. E.—(1928) *Brit. med. J.*, i, 494.
JACOBS, A.—(1960) *J. clin. Path.*, **13**, 463.—(1961*a*) *Ibid*, **14**, 610.—(1961*b*) *Brit. med. J.*, ii, 109.
JONES, A. M. AND OWEN, R. D.—(1928) *Ibid.*, i, 256.
KELLY, A. B.—(1919) *J. Laryng.*, **34**, 285.
KIRCHENBERGER, W. AND FLETT, R. L.—(1946) *Ibid.*, **61**, 396.
LEDERMAN, M.—(1958) *Ibid.*, **72**, 397.
MCGEE, L. C. AND GOODWIN, T. M.—(1938) *Ann. intern. Med.*, **11**, 1498.
MCGIBBON, J.—(1935) *J. Laryng.*, **50**, 329.
MOERSCH, H. J. AND CONNER, H. M.—(1926) *Arch. Otolaryng. Chicago.*, **4**, 112.
OWEN, R. D.—(1950) *Proc. roy. Soc. Med.*, **43**, 157.
PALMER, E. D.—(1952) ' The Oesophagus and its Diseases '. New York (P. B. Hoeber).
PATERSON, D. R.—(1919) *J. Laryng.*, **34**, 289.
SAVILAHTI, M.—(1946) *Acta med. scand.*, **125**, 40.
SHAMMA'A, M. H. AND BENEDICT, E. B.—(1958) *New Engl. J. Med.*, **259**, 378.
SIMPSON, R. R.—(1939) *J. Laryng.*, **54**, 738.
SUZMAN, M. M.—(1933) *Arch. intern. Med.*, **51**, 1.
WALDENSTROM,, J.—(1938) *Acta med. scand.*, Suppl. 90, 380.
WELIN, S.—(1953) *Brit. J. Radiol.*, **26**, 218.
WHITBY, L. AND BRITTON, C. J. C.—(1957) ' Diseases of the Blood ' 8th Ed. London (Churchill).
WILKINSON, J. F.—(1950) *Brit. med. J.*, ii, 576.
WISLOCKI, G. B., FAWCETT, D. W. AND DEMPSEY, E. W.—(1951) *Anat. Rec.*, **110**, 359.
WITTS, L. J.—(1931) *Guy's Hosp. Rep.*, **81**, 193.